# Research hotspots and trends in therapeutic drug monitoring of anticancer drugs: a 1990–2024 bibliometric analysis

**DOI:** 10.3389/fonc.2026.1617790

**Published:** 2026-03-31

**Authors:** Can Qian, Ting Yuan, Guanting Lu, Miao He, Xin Li, Huaiyu Su, Chenglong Li

**Affiliations:** 1 Department of Pharmacy, Deyang People’s Hospital, Deyang, China; 2 Deyang Key Laboratory of Tumor Molecular Research, Deyang People’s Hospital, Deyang, China; 3 Department of Oncology, Deyang People’s Hospital, Deyang, China

**Keywords:** anticancer drug, bibliometric analysis, chemotherapy drug, research hotspot, therapeutic drug monitoring, tyrosine kinase inhibitor

## Abstract

**Objective:**

To explore the current status of therapeutic drug monitoring (TDM) research on anticancer drugs, analyze research hotspots and trends, and provide insights and references for future studies.

**Methods:**

Data were retrieved from the Web of Science Core Collection (1990–2024) using keywords related to “anticancer drugs” and “therapeutic drug monitoring.” Bibliometric analyses were performed using VOSviewer, CiteSpace, R, and Scimago Graphica to visualize trends in publications, keywords, collaborations, and citation networks.

**Results:**

A total of 1474 articles were included. Global research output on TDM for anticancer drugs has grown steadily, with an accelerating trend in recent years. Key research themes include drug-specific monitoring, analytical methodologies, and clinical safety and efficacy. Busulfan remains the most studied agent in hematologic malignancies, while tyrosine kinase inhibitors, particularly crizotinib and nilotinib, along with monoclonal antibodies, have emerged as focal points of recent citation bursts. The keyword “pediatric patients” also shows a strong burst signal, reflecting growing attention to developmental pharmacokinetics and individualized dosing in vulnerable populations. The majority of influential studies were published in clinical oncology and pharmacology journals, with chemotherapy and targeted therapy dominating among the most cited papers. The United States, China, Japan, and Western European countries, notably the Netherlands and France, account for the majority of global publications. Among these, the Netherlands, Switzerland, and France demonstrate not only the highest research intensity, as measured by the Relative Importance Index (RII), but also a gradual increase in RII over time. Institutional-level RII analysis further highlights sustained contributions from leading European academic and clinical networks. International collaboration is highly concentrated among these high-output regions, forming a tightly interconnected research network.

**Conclusion:**

This study maps the evolving landscape of TDM in oncology. Accelerating research on tyrosine kinase inhibitors, monoclonal antibodies, and pediatric populations highlights TDM’s clinical value in optimizing therapy for narrow-therapeutic-index drugs. Rising research intensity in Europe and strong international collaboration underscore a coordinated global effort, supporting TDM’s integration into precision oncology, especially for vulnerable patients.

## 1 Introduction

Cancer poses a serious threat to human health, with high morbidity and mortality rates observed globally, and it also leads to significant economic and social problems (1). Drug therapy is one of the primary treatment modalities for cancer. Anticancer drugs, including chemotherapy agents, endocrine therapy drugs, molecular targeted drugs and immune checkpoint inhibitors (ICIs), exhibit complex pharmacokinetic properties and significant inter-individual variability (2–4). These factors lead to fluctuations in blood drug concentrations, making precise control essential to optimize efficacy and minimize safety risks (5–7).

Therapeutic drug monitoring (TDM) is a discipline dedicated to individualized dosing. It aims to optimize clinical outcomes by measuring blood drug concentrations and adjusting doses accordingly. Over the past years, TDM has gained increasing attention in the management of drugs that exhibit low therapeutic indices, narrow therapeutic windows, or a high risk of severe adverse reactions. It is also commonly used for drugs requiring long-term administration and those showing substantial variability between individuals. The research types encompass both clinical and basic studies. Currently, TDM is relatively more widely utilized in areas such as immunosuppressants (8, 9), antimicrobial agents (10), and antiepileptic drugs (11, 12).

In contrast, TDM in oncology was introduced at a later stage and remains limited in scope and clinical adoption (13, 14). Although used for certain agents since the 1970s, it is not universally applied, and therapeutic targets are not well established for most drugs (6).

Due to the typically prolonged courses of anticancer treatment, long-term medication may further exacerbate variations in drug exposure levels. Although there is a clear necessity for TDM of anticancer drugs, several challenges remain in practical implementation.

Common challenges apply broadly regardless of drug type. For instance, obtaining the area under the curve (AUC) for a single drug often requires multiple blood samples, which is highly inconvenient for patients (2). Meanwhile economic considerations also need to be taken into account. Additionally, some anticancer drugs are prodrugs, whose active metabolites are difficult to detect in the bloodstream (15). Furthermore, due to the unique blood supply of solid tumors, measuring the concentrations of the parent drug or its active metabolites in tumor tissues is extremely challenging (16–18).

Drug class-specific further complicate the implementation of TDM. In chemotherapy, regimens are typically based on combinations of drugs, making pharmacokinetic (PK)-pharmacodynamic (PD) relationships more difficult to model and target AUC values harder to define for individual agents (2). Tyrosine kinase inhibitors and anti-hormonal drugs typically require long-term daily administration, yet fixed dosing remains widely used. This is problematic because many of these agents exhibit considerable inter-individual variability in PK exposure and have a narrow therapeutic range. Moreover, well-defined exposure-response and exposure-toxicity relationships are lacking for some drugs at the approved dose, and suitable bioanalytical methods for monitoring are not always available (19). For ICIs, their PK and PD properties are fundamentally different from those of small-molecule drugs, which makes the development of TDM equally challenging (4). Nevertheless, researchers have conducted extensive exploration in the field of TDM for anticancer drugs. However, comprehensive analyses of the research hotspots and trends in this area are rarely reported.

Bibliometrics is a quantitative analysis tool that reveals hot topics and trends in scientific research (20). By systematically analyzing large volumes of literature, it identifies high-impact keywords, core authors, international collaboration networks, key research institutions and technologies. This study employs bibliometric methods to explore the advances in TDM for anticancer drugs globally since 1990, analyze research hotspots and trends, and provide references for researchers.

## 2 Materials and methods

### 2.1 Data collection

**Figure 1:**
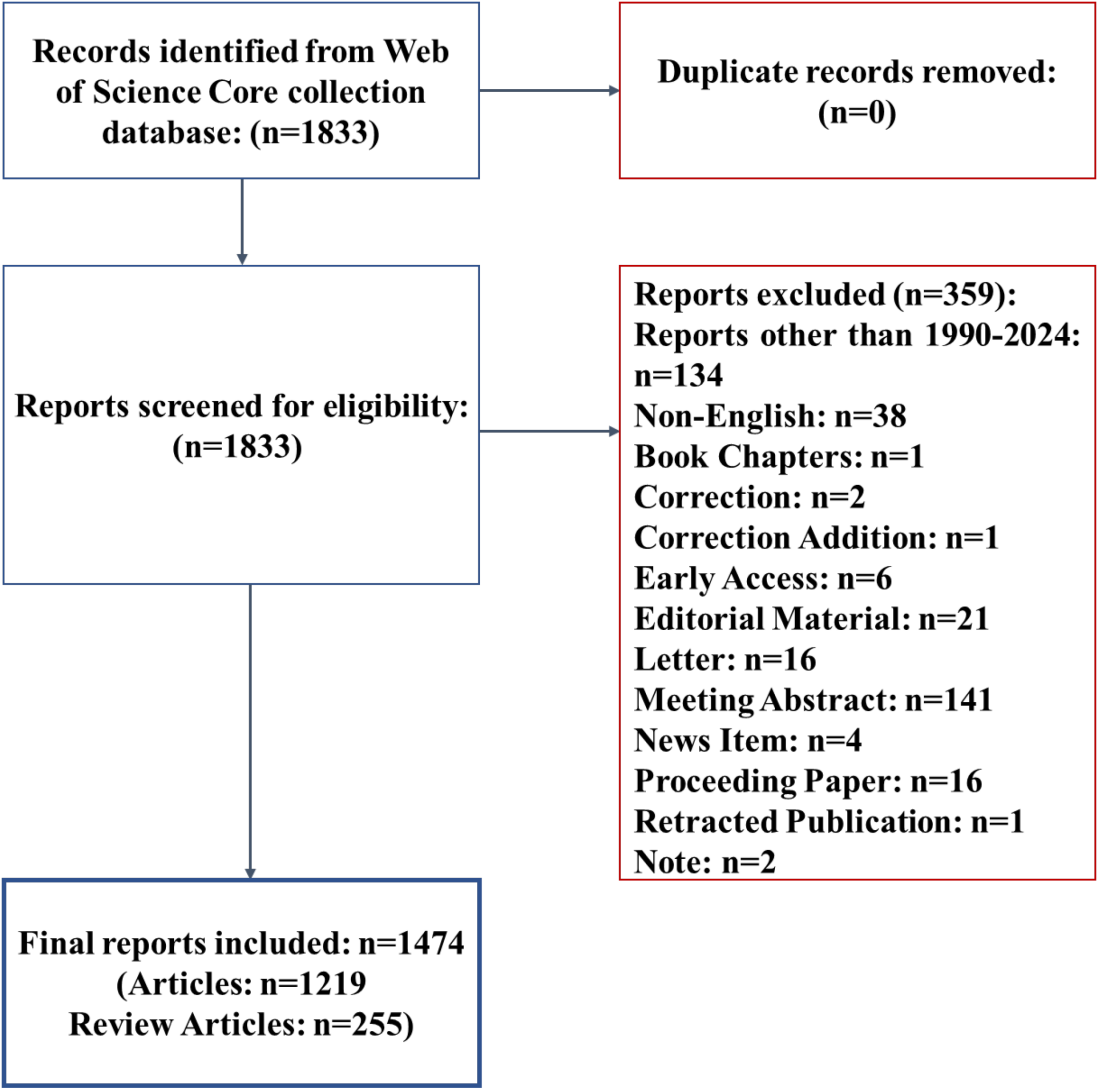
Flow chart outlining the steps for study inclusion.

Web of Science (WOS) is widely recognized as a comprehensive and reliable database for bibliometric analysis, particularly well-suited for global-scale research. With coverage of over 12000 influential high-quality journals worldwide it is regarded as one of the most authoritative resources for scientific publication analysis. The Web of Science Core Collection (WOSCC) has been consistently identified in previous studies as the preferred repository for bibliometric investigations (10, 21, 22). The reference data on TDM and anticancer drugs were retrieved from the Science Citation Index Expanded (SCI-E) database through the WOSCC using a topic-based search strategy. The original data of full records and cited references included elements such as titles, authors, source titles, abstracts, publication dates, keywords, affiliations, countries, and more. The analysis covered the period from 1990 to 2024. Only publications in English were included, and the following document types were excluded: book chapters, corrections, early access articles, editorial materials, letters, meeting abstracts, news items, proceeding papers, retractions, and notes. A total of 1474 articles were involved in this study from Jan 1990 to Dec 2024 (**Figure 1**).

The advanced database search strategy included specific terms related to anticancer drugs and TDM (see **Supplementary Material**).

### 2.2 Data analysis and visualization

A multi-software approach was employed for bibliometric analysis and visualization. VOSviewer (version 1.6.20) and CiteSpace (version 6.4.R1, Advanced) were used for network construction and thematic mapping, while R (version 4.3.2) and SCImago Graphica (version 1.0.51) were utilized for the visualization of specific datasets.

The VOSviewer software was utilized to analyze and visualize keyword co-occurrence networks, author collaboration relationships, and citation co-occurrence patterns. To construct the maps, VOSviewer employs the visualization of similarities (VOS) mapping technique, where VOS stands for Visualization of Similarities (23). In essence, the VOS algorithm optimizes the distances between nodes so that their positions in a two-dimensional space intuitively reflect the strength of their associations, thereby providing a clear visualization of scientific knowledge networks (24).

CiteSpace software (25) was employed to identify emerging research trends and analyze thematic evolution. Keyword bursts were detected using the Kleinberg algorithm, which identifies sudden increases in term frequency by modeling them as state transitions over time (26). Thematic evolution was analyzed via dual-map overlay, which visualizes citation flows between Web of Science journal categories by mapping the sources and targets of citations, thereby revealing the interdisciplinary knowledge origins and impacts of the research domain (27).

In addition, R and Scimago Graphica were employed to enhance the clarity and visualization of the most prominent drug-specific keywords related to anticancer drugs in TDM studies, as well as country and institutional collaboration networks.

The Relative Importance Index (RII) was used to evaluate the research intensity of countries and institutions. The calculation method is set as follows:


$$\text{RII}=100000\times \frac{\text{Number of publications on anticancer drug TDM}\;}{\text{Total number of publications}}$$

## 3 Results

### 3.1 Overview of annual publication number

**Figure 2:**
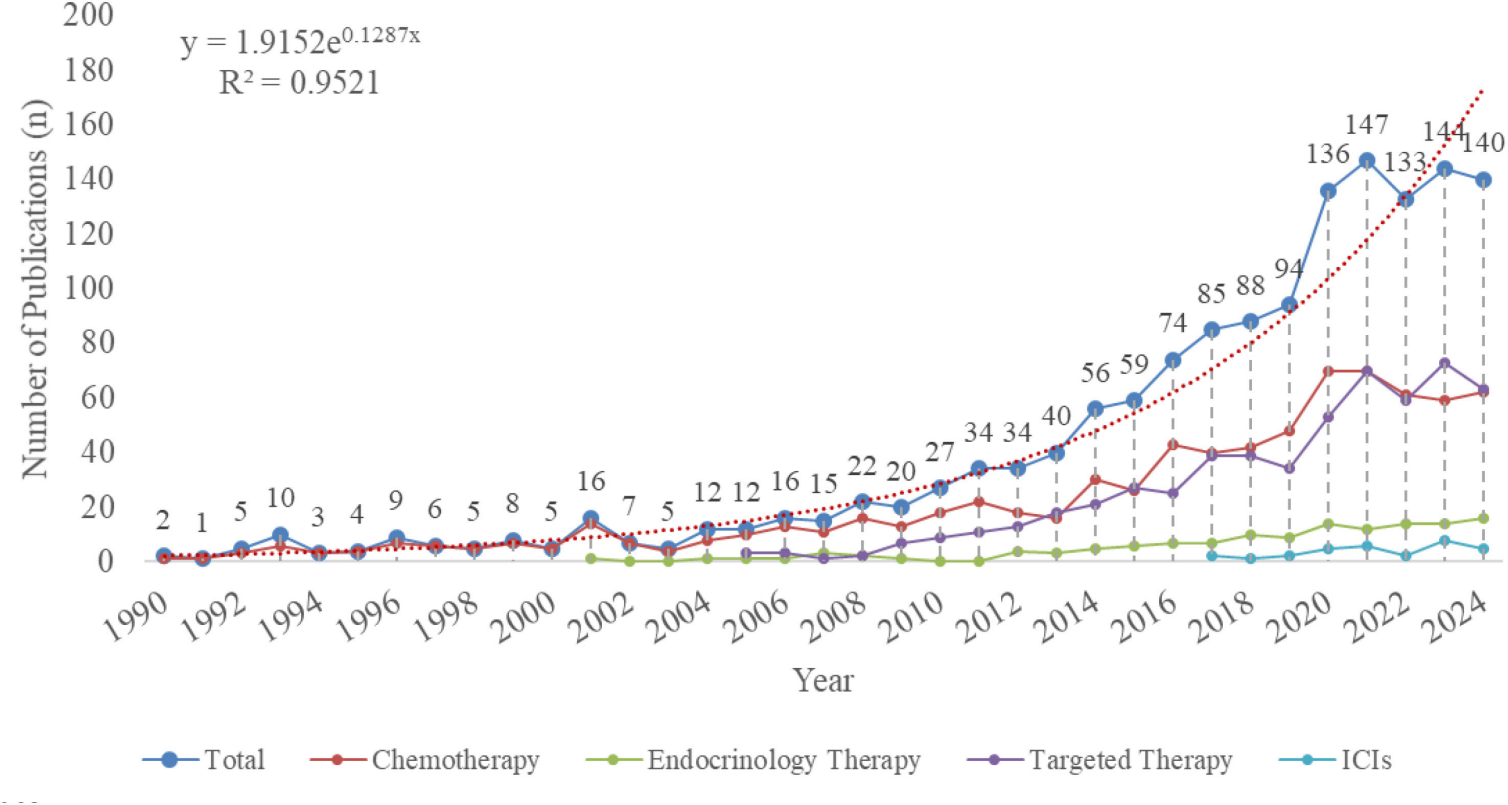
Annual publication number in the field of TDM in anticancer drugs from 1990 to 2024. The red dashed line represents the exponential trendline fitted using the least squares method.

There has been a substantial increase in academic activity and influence within this field (**Figure 2**). The annual number of publications increased from 2 in 1990 to 140 in 2024, showing an overall growth trend. However, since 2020, the growth rate has temporarily slowed down. The highest number of articles, 147 in total, were published in 2021. Among all the included literature, research articles accounted for 83%, with the remainder being review articles. **Figure 2** also sheds light on the temporal trends and onset of TDM research across four major classes of anticancer drugs, with different colors representing each category.

### 3.2 Specific drug types

**Figure 3:**
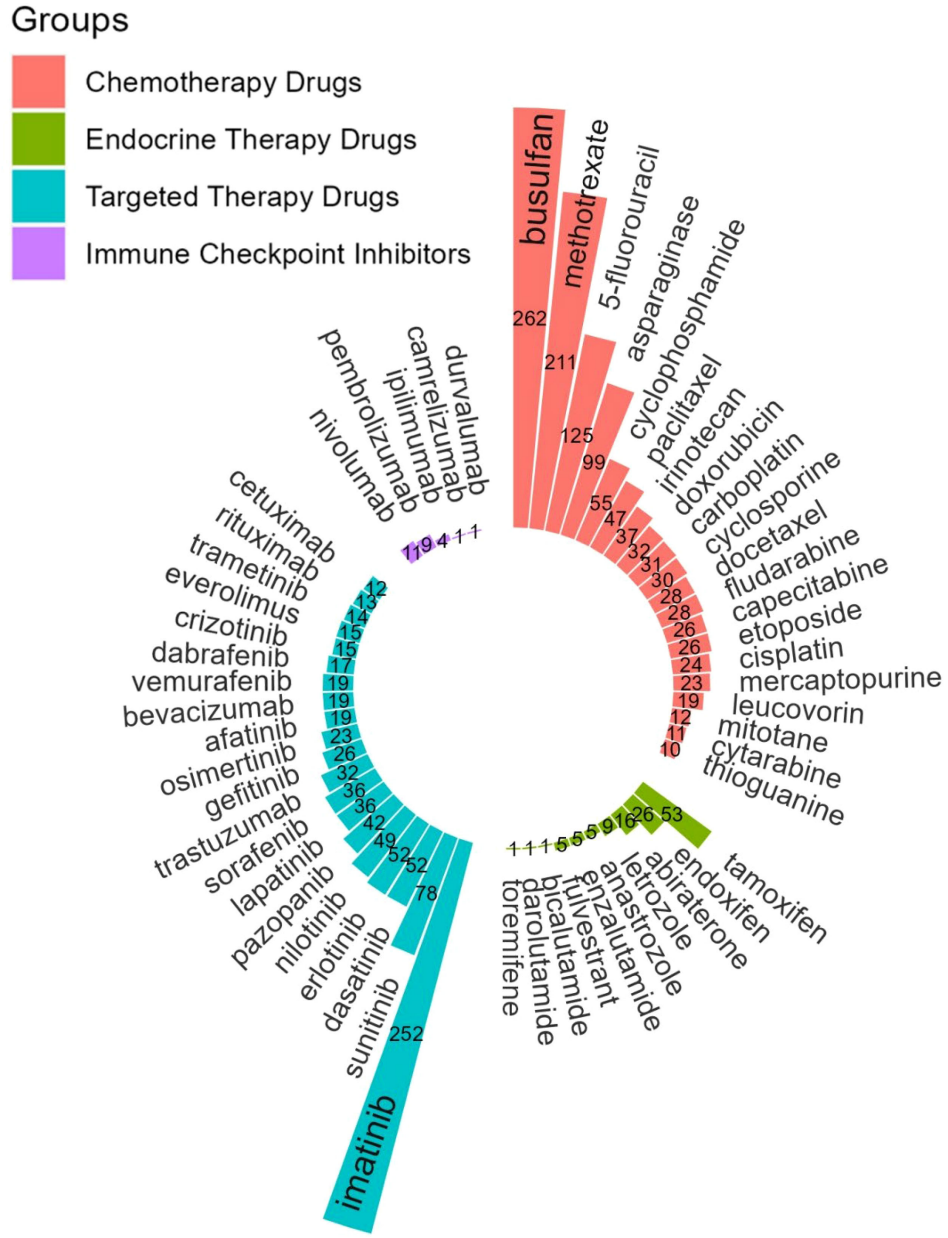
Top 20 drug-specific keywords screened from the keyword co-occurrence network of anticancer drugs in TDM studies.

By extracting keywords from the literatures, we identified that specific drug species were mentioned 2386 times. These drugs are mainly categorized into chemotherapy drugs, targeted therapy drugs, endocrine therapy drugs, and ICIs. In **Figure 3**, we screened and listed the top 20 most frequently mentioned specific drugs in each category (all drugs are listed if fewer than 20 distinct types are present). Among these, busulfan was the most frequently mentioned drug in the keywords, appearing 262 times, followed by imatinib (n = 252), methotrexate (n = 211), 5-fluorouracil (n = 125), and asparaginase (n = 99).

### 3.3 Keyword co-occurrence and clustering

**Figure 4:**
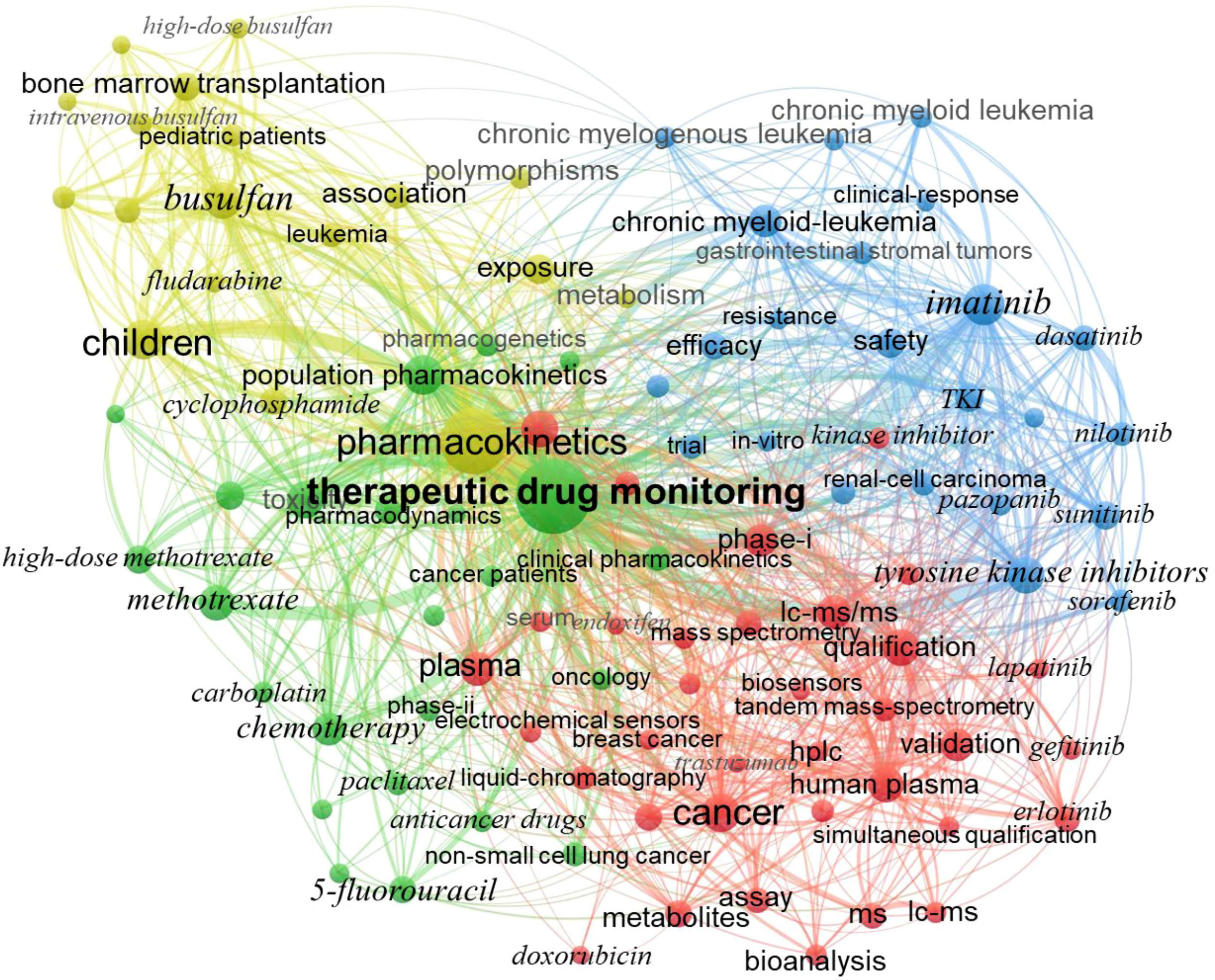
Keyword co-occurrence and clustering. The size of nodes and text corresponds to keyword co-occurrence frequency, connecting lines represent co-occurrence relationships, and nodes sharing the same color belong to the same cluster.

A total of 4907 keywords were identified across all the literature. Keywords with a frequency of ≥ 25 were selected for clustering and co-occurrence analysis based on their frequency and category. This process yielded the top 100 high-frequency keywords, whose relationships are illustrated in **Figure 4**. The top five most frequently occurring keywords are “therapeutic drug monitoring” (n = 671), “pharmacokinetics” (n = 497), “tyrosine kinase inhibitors” (TKIs, n = 325), “imatinib” (n = 252), and “children/pediatric patients” (n = 246). Overall, the research is primarily centered around TDM and PK. Upon closer examination, the studies can be divided into four main categories:

The yellow cluster focuses on drug exposure in special populations and clinical applications in hematological diseases, including pediatric patients, busulfan, bone marrow transplantation, cyclophosphamide, and genetic polymorphisms. The inclusion of pediatric patients in this cluster reflects the clinical recognition of heightened PK variability and increased vulnerability to toxicity in children, making TDM particularly relevant for optimizing dosing in this population.

The blue cluster is dominated by TKIs, including imatinib, dasatinib, and sunitinib, as well as related diseases such as leukemia. This clustering underscores the clinical importance of TDM for TKIs, which exhibit high interpatient variability and are used in chronic settings where sustained drug exposure is critical to prevent resistance.

The red cluster emphasizes quantitative detection methods, featuring techniques such as liquid chromatography, mass spectrometry, liquid chromatography-mass spectrometry (LC-MS), and tandem mass spectrometry (LC-MS/MS), and even emerging technologies such as electrochemical sensors and biosensors. The prominence of analytical methods in this cluster highlights that accurate and reliable drug measurement is the foundational step for any TDM practice, enabling precise dose individualization in clinical decision-making.

The green cluster concentrates on chemotherapy drugs and population PKs, including high-dose methotrexate, paclitaxel, and 5-fluorouracil. This cluster reflects the long-standing need for TDM with conventional chemotherapeutics that have narrow therapeutic windows and highly variable PKs, where population models help guide dosing in diverse patient groups.

These four clusters are not completely independent but rather exhibit significant overlap. Some keyword nodes at the boundaries of clusters may belong to two categories simultaneously. For example, erlotinib, although classified as a TKI, is associated with numerous studies on detection methods, resulting in its placement in the red cluster. Additionally, some highly related keywords, though assigned to different clusters, demonstrate strong connections, such as “children”, “high-dose methotrexate”, “leukemia”, and “busulfan”. This interconnectedness highlights the multidisciplinary nature of the research and underscores the complex relationships between various fields within the study.

### 3.4 Keyword burst analysis

**Figure 5:**
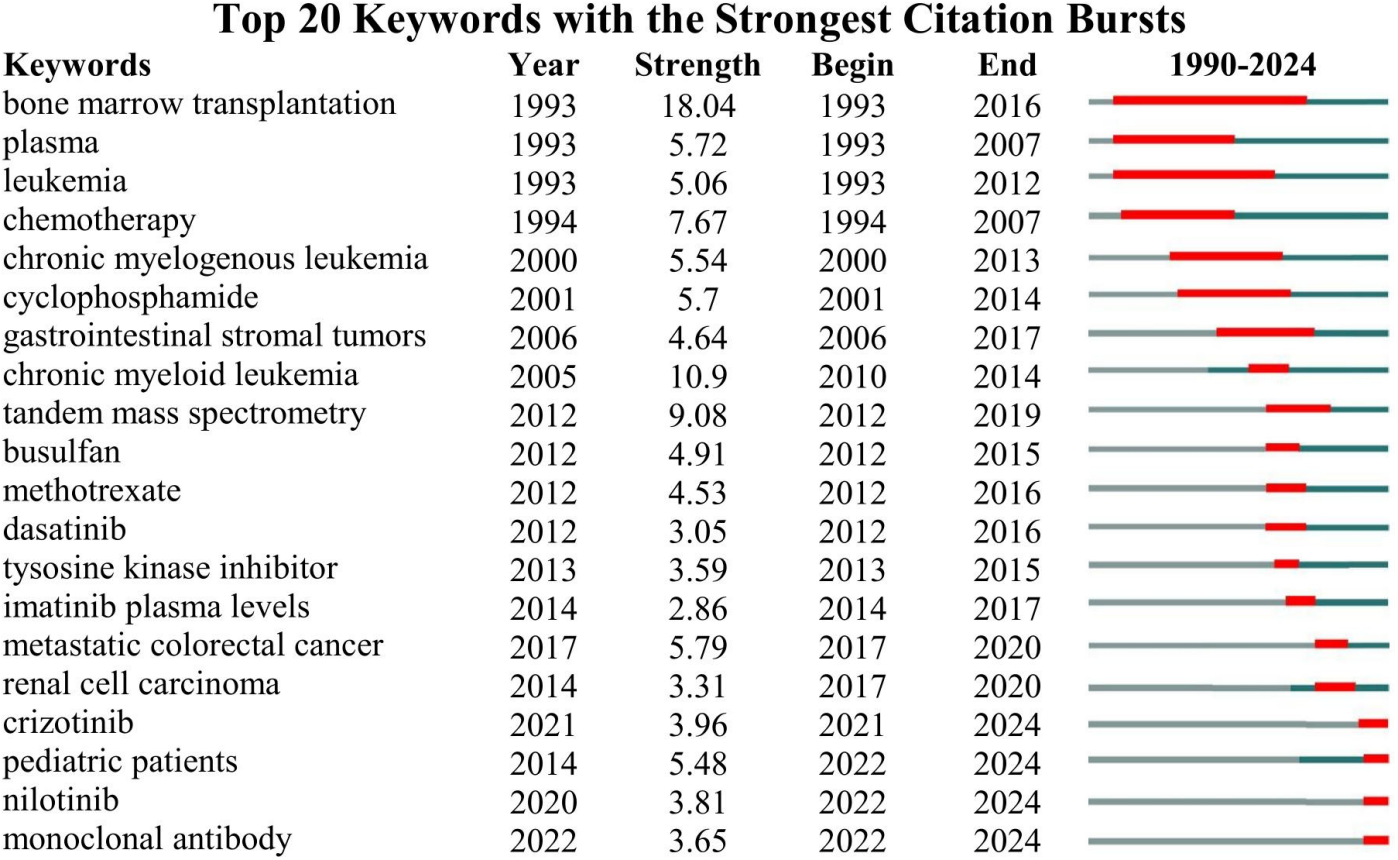
Top 20 keywords with the strongest citation bursts. For each keyword, a red band indicates a strong burst, a dark green band represents a weak but still significant burst, and a gray band signifies no burst.

The keyword burst detection function in CiteSpace can be used to reflect the primary research hotspots, developmental trends, and frontier dynamics within a specific field over a given period. By setting a threshold (a minimum burst duration of three years), we identified the top 20 research hotspot keywords in its certain period, as shown in **Figure 5**. The results reveal 20 major research hotspots in the field of TDM for anticancer agents have exhibited bursts from 1990 to 2024, indicating that scholars continue to actively engage in these areas. Notably, “leukemia”, “bone marrow transplantation” and “plasma” demonstrated strong bursts shortly after the study period began, with activity emerging as early as 1993. Among these, “bone marrow transplantation” exhibited a strong burst until the end of 2016 and maintained a weak burst status until 2024, making it the keyword with the highest burst strength (strength = 18.04) among the 20 keywords. After 2000, new research hotspots emerged, and by 2022, these hotspots encompassed drugs, diseases, and analytical methods. As of the end of 2024, keywords that have recently emerged as research hotspots include “crizotinib”, “pediatric patients”, “nilotinib”, and “monoclonal antibody”. These not only represent the research frontier but also illustrate the expanding focus on a broader range of targeted therapies and special populations, such as pediatric patients.

### 3.5 Primary publishing journals, co-cited journals, most cited articles, and thematic evolution analysis via dual-map overlay

In **Table 1A**, **1B**, and **1C**, we present the top 11 (including ties) publishing journals, the top 10 co-cited journals, and the top 10 most cited articles in the context of TDM for anticancer drugs. Within the top 10 publishing journals, *Therapeutic Drug Monitoring* leads with the highest publication count, representing 10.52% (155/1474) of the total, while *European Journal of Cancer* holds the highest impact factor (IF = 7.1). For the top 10 co-cited journals, all have been co-cited over 1000 times. Notably, *Journal of Clinical Oncology* ranks highest in citations (n = 3341), whereas *New England Journal of Medicine* boasts the highest impact factor (IF = 78.5). The top 10 most cited articles mainly focus on chemotherapy and targeted drugs.

**Table 1A:** Top 11 journals publishing on TDM of anticancer drugs (including ties).

Rank	Journal	Documents	IF (2024)	JCR
1	Therapeutic Drug Monitoring	155	2.4	Q2
2	Journal of Pharmaceutical and Biomedical Analysis	67	3.1	Q2
3	Cancer Chemotherapy and Pharmacology	55	2.3	Q3
4	Journal of Chromatography B-analytical Technologies in the Biomedical and Life Sciences	53	2.8	Q2
5	British Journal of Clinical Pharmacology	48	3.0	Q2
6	Clinical Pharmacokinetics	38	4.0	Q2
7	Biomedical Chromatography	31	1.7	Q3
8	Journal of Clinical Pharmacology	19	2.3	Q3
9	European Journal of Cancer	17	7.1	Q1
9	Pharmaceutics	17	5.5	Q1
9	Expert Opinion on Drug Metabolism & Toxicology	17	3.4	Q1

**Table 1B:** Top 10 co-cited journals in TDM research on anticancer drugs.

Rank	Cited Journal	Co-citation	IF (2024)	JCR
1	Journal of Clinical Oncology	3341	41.9	Q1
2	Cancer Chemotherapy and Pharmacology	2110	2.3	Q3
3	Journal of Chromatography B-analytical Technologies in the Biomedical and Life Sciences	1976	2.8	Q2
4	Therapeutic Drug Monitoring	1848	2.4	Q2
5	Blood	1649	23.1	Q1
6	Clinical Pharmacokinetics	1618	4.0	Q2
7	Clinical Cancer Research	1579	10.2	Q1
8	Clinical Pharmacology & Therapeutics	1559	5.5	Q1
9	New England Journal of Medicine	1298	78.5	Q1
10	Bone Marrow Transplantation	1195	5.2	Q1

**Table 1C:** Top 10 most cited papers.

Rank	Year	Author	Counts	Article title	Journal
1	1996	Gurney H	289	Dose calculation of anticancer drugs: A review of the current practice and introduction of an alternative	Journal of Clinical Oncology
2	2009	Zhou SF	286	Polymorphism of Human Cytochrome P450 2D6 and Its Clinical Significance Part II	Clinical Pharmacokinetics
3	2019	Wenningmann N	263	Insights into Doxorubicin-induced Cardiotoxicity: Molecular Mechanisms, Preventive Strategies, and Early Monitoring	Molecular Pharmacology
4	2014	Widmer N	275	Review of therapeutic drug monitoring of anticancer drugs part two-targeted therapies	European Journal of Cancer
5	2017	Verheijen RB	226	Practical Recommendations for Therapeutic Drug Monitoring of Kinase Inhibitors in Oncology	Clinical Pharmacology & Therapeutics
6	2016	Bartelink IH	223	Association of busulfan exposure with survival and toxicity after haemopoietic cell transplantation in children and young adults: a multicentre, retrospective cohort analysis	Lancet Haematology
7	2014	Paci A	218	Review of therapeutic drug monitoring of anticancer drugs part 1-Cytotoxics	European Journal of Cancer
8	2014	Yu HX	209	Practical guidelines for therapeutic drug monitoring of anticancer tyrosine kinase inhibitors: focus on the pharmacokinetic targets.	Clinical Pharmacokinetics
9	2019	Beumer JH	94	Therapeutic Drug Monitoring in Oncology: International Association of Therapeutic Drug Monitoring and Clinical Toxicology Recommendations for 5-Fluorouracil Therapy	Clinical Pharmacology & Therapeutics
10	2017	Huynh HH	81	Development and Validation of a Simultaneous Quantification Method of 14 Tyrosine Kinase Inhibitors in Human Plasma Using LC-MS/MS	Therapeutic Drug Monitoring

**Figure 6:**
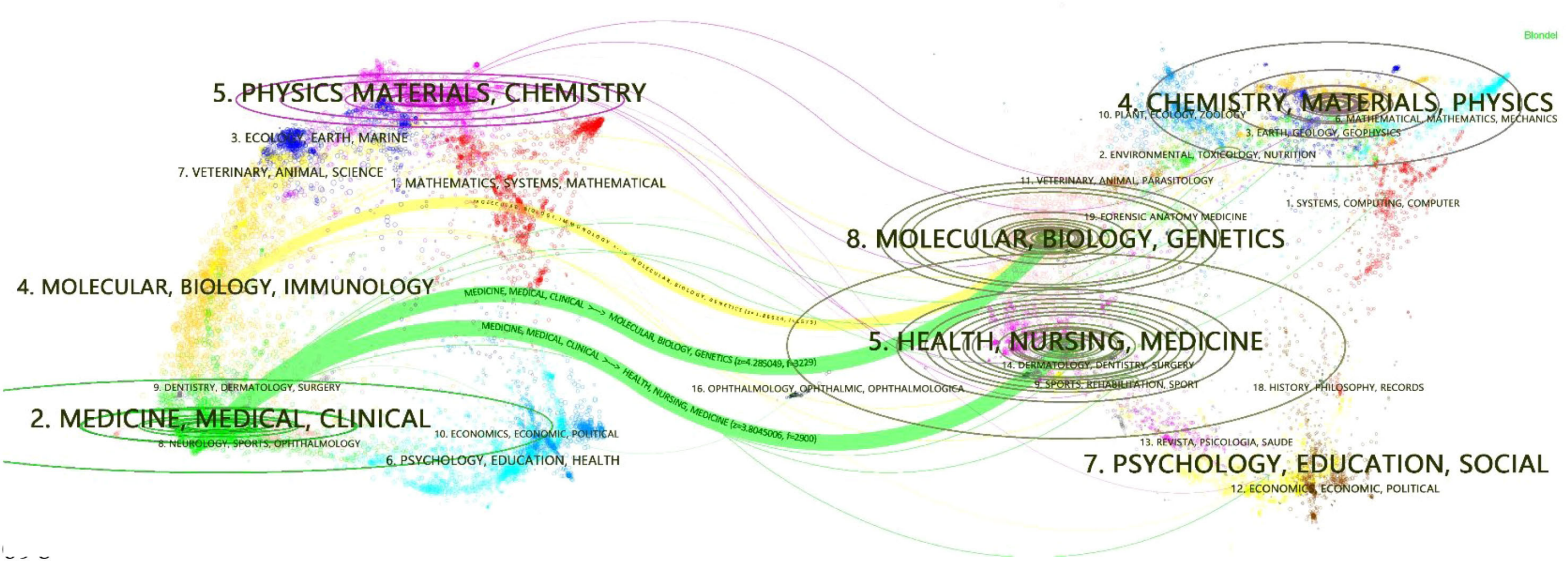
Double-map coverage of journals related to anticancer drugs in TDM research. Citing journals (left) represent the knowledge frontier, and cited journals (right) indicate foundational literature. Labels denote journal-covered topics; colored paths represent citation relationships between labels. Ellipses signify topic clusters; their vertical position correlates positively with paper counts, and horizontal position reflects author numbers.

The development trajectory of disciplinary topics can be illustrated through a dual map overlay (**Figure 6**). Key citation paths include two green and one yellow path. The green paths depict a “convergent” model where research in Molecular/Biology/Immunology and Health/Nursing/Medicine fields is cited by Medicine/Medical/Clinical journals. The yellow path shows a “divergent” pattern, with Molecular/Biology/Genetics research cited by Molecular/Biology/Immunology journals. Additionally, although the Physics/Materials/Chemistry field lacks major connecting paths, it exhibits clustering developed from the convergence of multiple topics on the right side.

### 3.6 Author analysis

**Figure 7:**
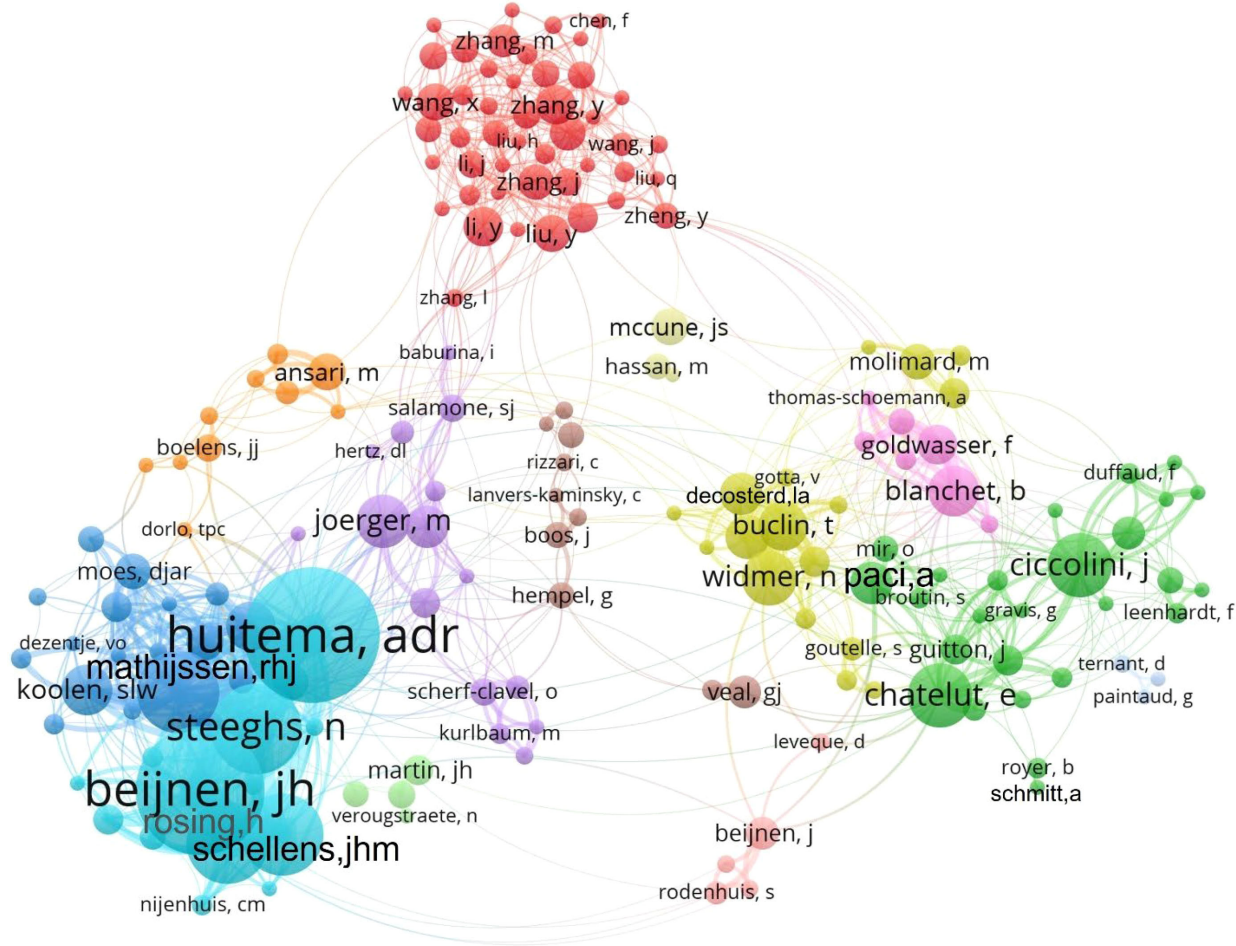
Author collaboration network in TDM research of anticancer drugs. The size of nodes and text corresponds to the number of publications by each author, connecting lines represent co-authorship relationships, and nodes sharing the same color belong to the same collaboration cluster.

**Figure 8:**
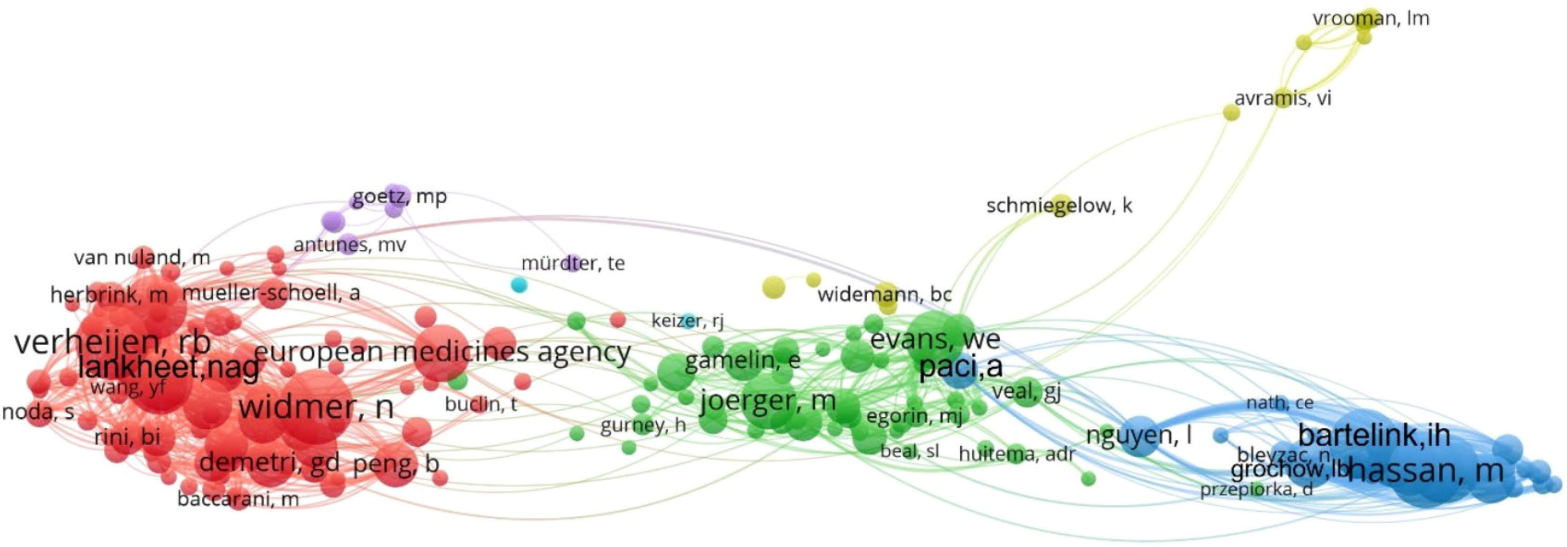
Co-citation network of authors in TDM research of anticancer drugs. The size of nodes and text corresponds to the co-citation frequency of each author, connecting lines represent co-citation relationships, and nodes sharing the same color belong to the same intellectual cluster.

The author collaboration network and co-citation relationships are shown in **Figure 7** and **8**. The top three authors by publication count are Huitema Alwin DR, Beijnen Jos H, and Steeghs N, whose research primarily focuses on TKI-related TDM. The most frequently co-cited authors is Widmer N who specializes in population pharmacokinetics and personalized precision medicine. The second most co-cited author is Verheijen RB, whose work centers on oncology, pharmacology, and medical experimental techniques. The third most co-cited author is Bartelink IH, who has contributed to research on personalized dosing strategies for anticancer drugs in hematology. Additionally, the visualization maps provide insights into potential collaborations among researchers as well as thematic clusters within the field.

### 3.7 National collaboration network and research intensity

**Figure 9:**
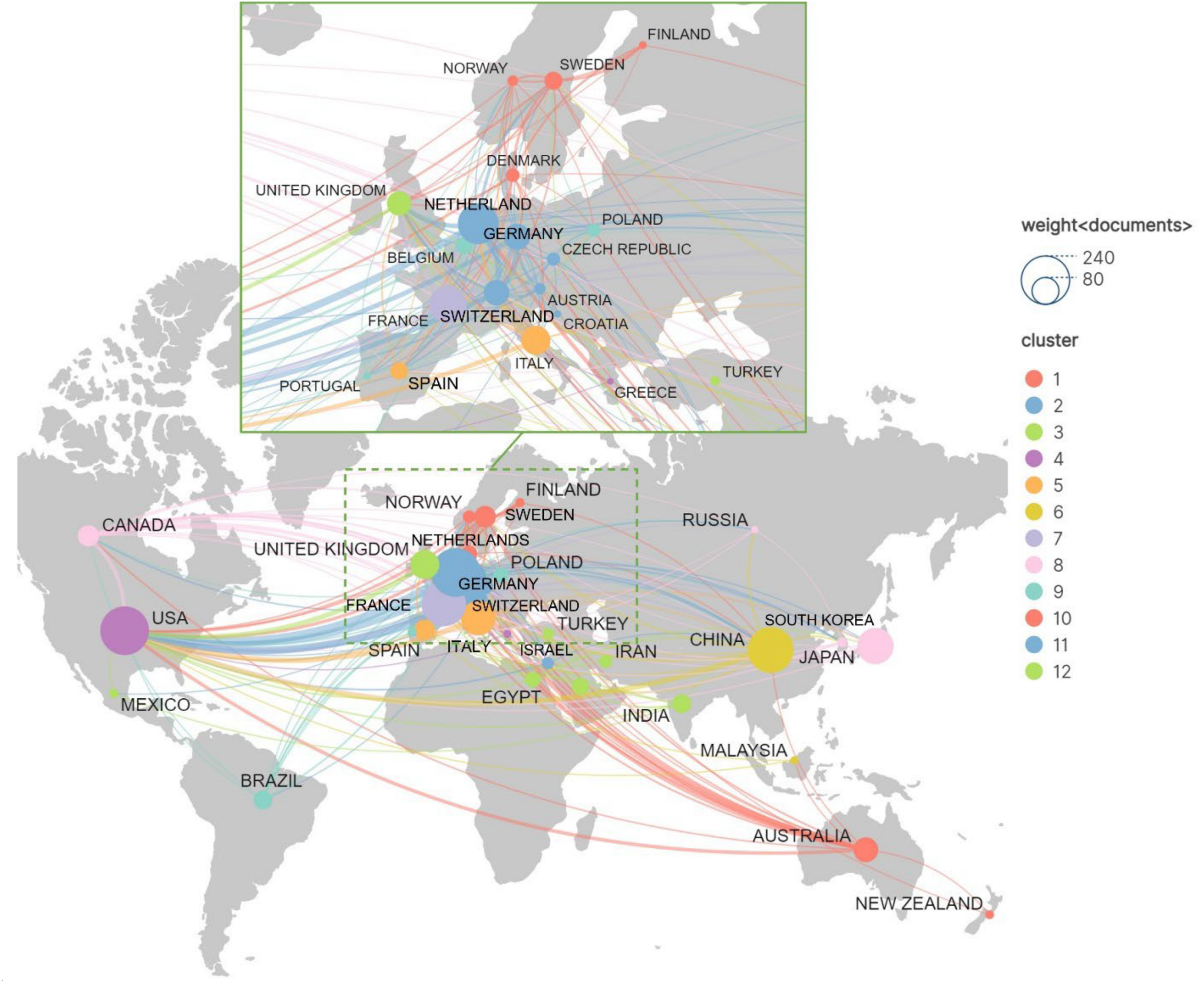
National collaboration network.

**Figure 10:**
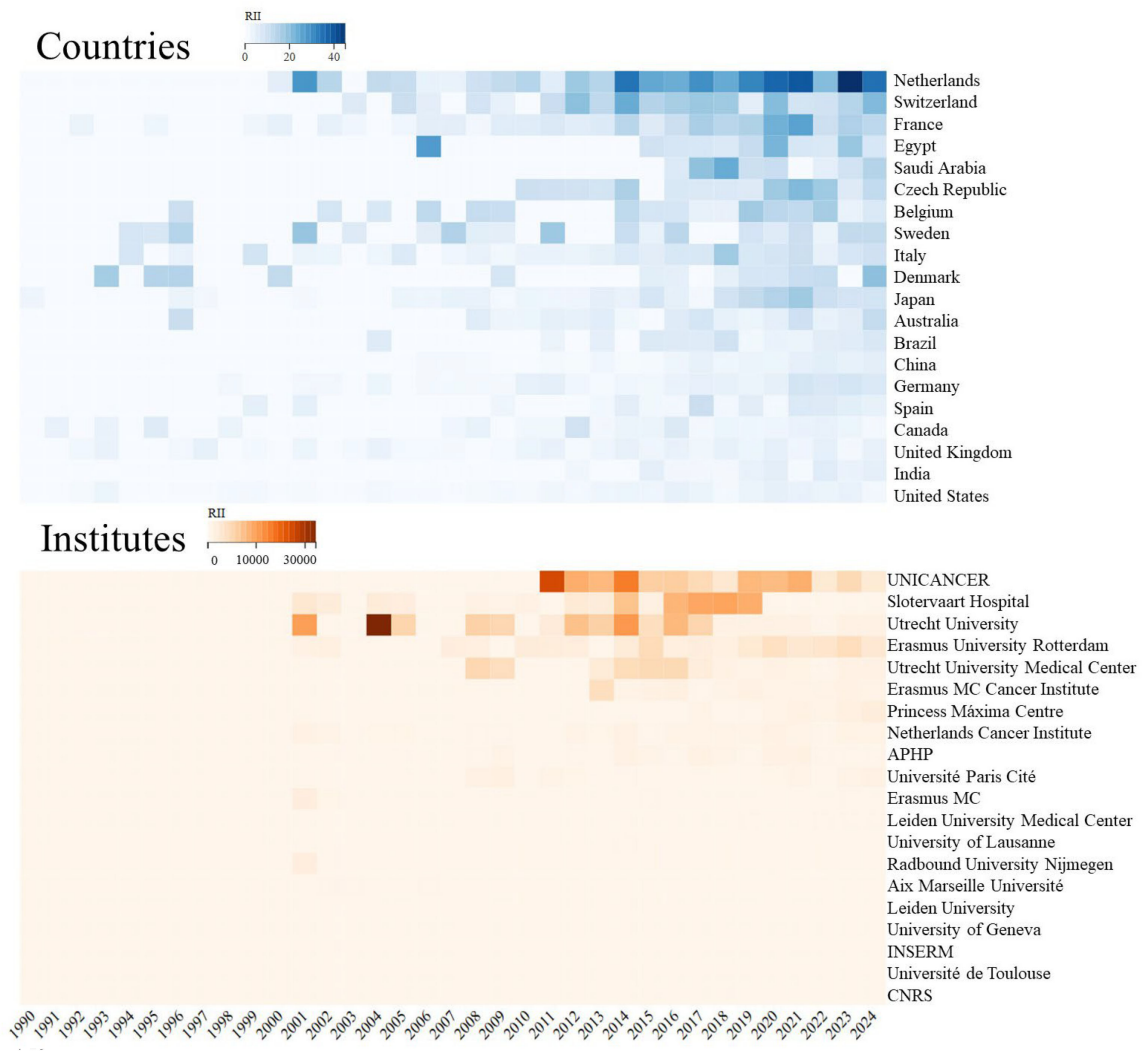
Heatmap of annual research intensity trends for countries and institutions. The heatmap is based on annually RII data. Countries and institutions are represented using different color schemes and scales. Within each color scheme, darker shades indicate higher RII values, reflecting greater research intensity.

The analysis of research publication quantity and collaboration among different countries in the field of TDM for anticancer drugs is as shown in **Figure 9**. By setting a minimum publication threshold of 5 articles per country, we selected the top 35 countries based on their total number of publications. Visualization was performed using VOSviewer in conjunction with ScimagoGraphica software. The size of each node reflects the number of articles published by that country, while the lines connecting the nodes represent collaborations between countries. Thicker lines indicate more frequent collaborations, and the color of the nodes represents different clusters of countries with similar characteristics. This figure highlights the close collaboration among high-publication countries. The Netherlands, the United States, China, France, and Japan stand out in the graph, representing their relatively high publication volumes. The United States leads in the number of both publications and citations (**Table 2**) and has particularly strong connections with other countries. There are also robust internal collaborations among European countries. The Relative Importance Index (RII) was used to evaluate the research intensity of leading countries in the field of TDM for anticancer drugs (**Supplementary Material**). Three European countries—the Netherlands, Switzerland, and France—ranked highest (**Table 3**), and their research output showed a gradual increase over time (**Figure 10**).

**Table 2:** Top 10 countries and institutions by publication volume.

Rank	Country	Publication	Citation	Institution	Publication	Centrality
1	USA	225	7709	Utrecht University	97	0.05
2	Netherlands	221	6873	Netherlands Cancer Institute	92	0.01
3	China	195	2348	Institut National de la Santé et de la Recherche Médicale (INSERM)	95	0.02
4	France	184	5756	UNICANCER	75	0.05
5	Japan	122	2037	Erasmus MC	59	0.01
6	Italy	111	3022	Erasmus University Rotterdam	59	0.01
7	Germany	92	2599	Utrecht University Medical Center	58	0.05
8	Switzerland	87	3660	Assistance Publique - Hôpitaux de Paris (APHP)	55	0.12
9	UK	87	2983	Université Paris Cité	41	0.1
10	Australia	58	1922	Erasmus MC Cancer Institute	41	0.02

**Table 3:** Top 20 countries and institutions by research intensity.

Rank	Country	Research intensity	Institution	Research intensity
1	Netherlands	18.41	UNICANCER	7883.82
2	Switzerland	9.38	Slotervaart Hospital	2021.13
3	France	7.56	Utrecht University	1396.61
4	Egypt	7.37	Erasmus University Rotterdam	874.71
5	Saudi Arabia	7.18	Utrecht University Medical Center	866.23
6	Czech Republic	6.94	Erasmus MC Cancer Institute	816.80
7	Belgium	5.93	Princess Maxima Center	711.74
8	Sweden	5.39	Netherlands Cancer Institute	423.56
9	Italy	5.08	Assistance Publique - Hôpitaux de Paris (APHP)	357.67
10	Denmark	4.38	Université Paris Cité	153.79
11	Japan	3.99	Erasmus MC	83.16
12	Australia	3.61	Leiden University Medical Center	70.59
13	Brazil	3.00	University of Lausanne	46.23
14	China	2.62	Radboud University Nijmegen	41.58
15	Germany	2.61	Aix Marseille Université	40.74
16	Spain	2.42	Leiden University	39.71
17	Canada	1.98	University of Geneva	34.55
18	United Kingdom	1.94	Institut national de la santé et de la recherche médicale (INSERM)	27.12
19	India	1.85	Université de Toulouse	24.81
20	USA	1.54	Centre national de la recherche scientifique (CNRS)	4.73

### 3.8 Institutional collaboration network and research intensity

**Figure 11:**
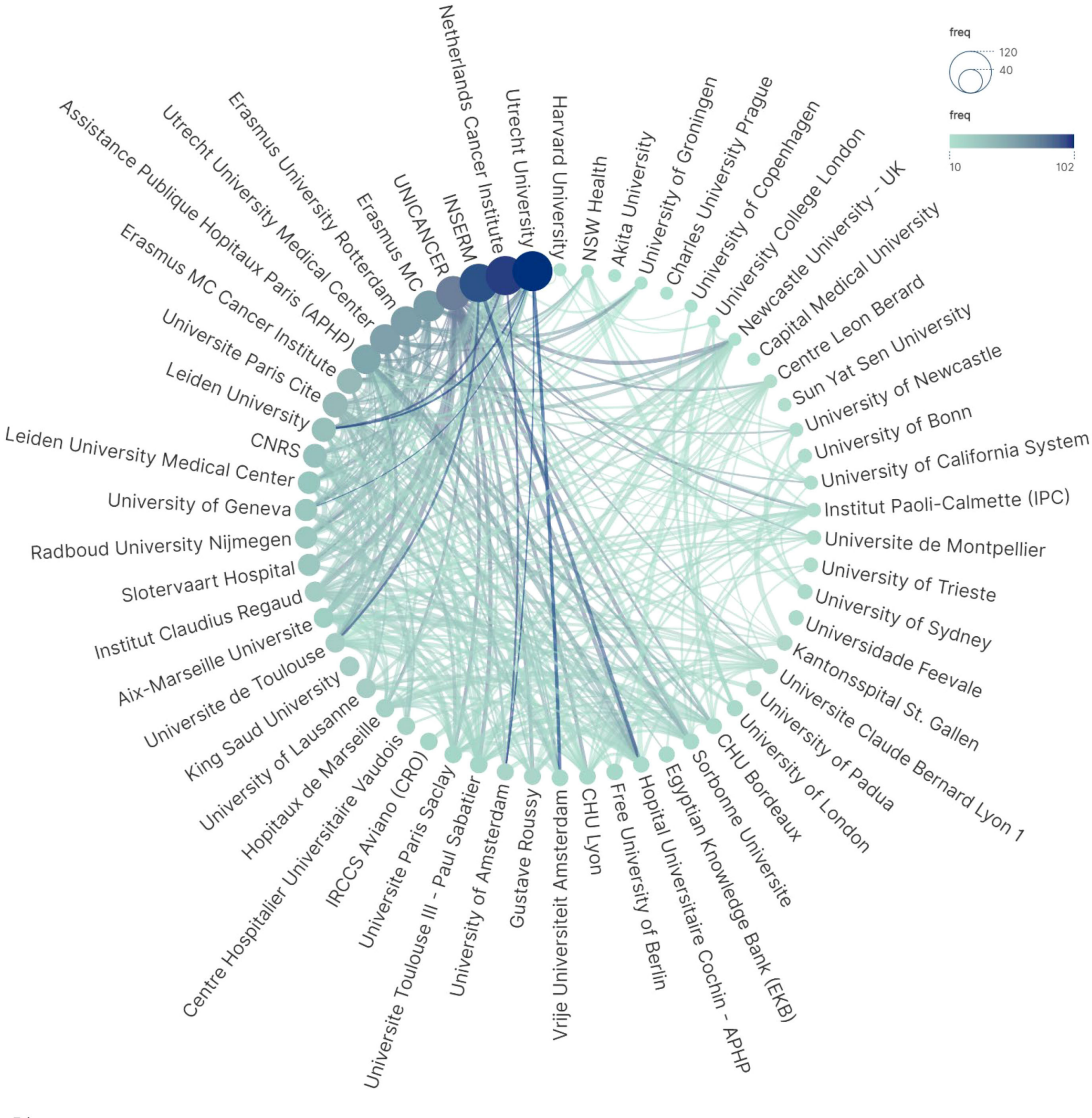
Institutional collaboration network.

The institutional collaboration network not only reflects the strength of national collaborations but also highlights the level of engagement of specific research institutions within the field (**Figure 11**). We selected the top 58 institutions with a minimum of 10 publications for the collaboration network analysis. Institutions are represented by nodes sized according to their publication volume, from largest to smallest, with larger and darker nodes indicating higher publication counts. Lines connecting the nodes represent collaborations between institutions, with thicker lines denoting stronger collaboration intensity. The institutions are predominantly universities, hospitals, and cancer research institutes. Among the top ten institutions with the highest individual publication counts, all are based in France and the Netherlands (**Table 2**). The top five are Utrecht University (Netherlands), Institut National de la Santé et de la Recherche Médicale (INSERM, France), Netherlands Cancer Institute, The French Comprehensive Cancer Centres’ Network (UNICANCER), and Erasmus University Rotterdam (Netherlands). These five institutions exhibit strong internal collaborations within their respective countries and demonstrate significant international engagement and influence. Likewise, the RII was applied to assess the research intensity of these institutions. UNICANCER, the French hospital federation, ranked first, followed by two institutions from the Netherlands: Slotervaart Hospital and Utrecht University (**Table 3**, **Figure 10**). However, the temporal trend in institutional research intensity was less pronounced compared to that observed at the country level.

## 4 Discussion

Since its introduction into clinical practice in the early 1960s, TDM has seen a rapid increase in research activity, primarily in non-oncological therapeutic areas. However, its application in oncology remained very limited until the 1980s (13, 14). Routine clinical use was confined to methotrexate, which was then predominantly employed in the treatment of diseases such as acute lymphoblastic leukemia (ALL) (14). For instance, Evans et al. (28). established the relationship between systemic methotrexate clearance and the probability of relapse in children with ALL. Additionally, approximately one-third of the studies focused on bioavailability assessment, primarily involving mercaptopurine and melphalan (29, 30). For other agents such as cytarabine (Ara-C), doxorubicin, and cyclophosphamide, research was still in the stage of pharmacokinetic investigation and preclinical or early clinical data accumulation (31–33).

Since then, the emergence of new technologies and therapeutics, along with a deeper understanding of cancer and anticancer drugs, has gradually expanded the role of TDM in oncology. This study used bibliometric methods to map and describe the research hotspots and trends in the field of TDM for anticancer drugs globally between 1990 and 2024. Over the past 35 years, the number of publications worldwide in this field has steadily risen. Despite rapid progress in oncology, the relative focus on TDM for anticancer drugs has steadily increased over time, reflecting growing interest in optimizing therapeutic outcomes through individualized monitoring.

Keyword analysis employed co-occurrence, clustering, and burst detection. The most frequent terms were “therapeutic drug monitoring” and “pharmacokinetics.” TDM is crucial for optimizing outcomes and minimizing toxicity in anticancer therapy due to interpatient variability in drug metabolism. Although pharmacokinetic studies are common in early drug development, their clinical relevance remains high, especially for personalized dosing. The expanding use of targeted therapies and immunotherapies further underscores the importance of PK research.

The research in this field has largely revolved around three main categories related to TDM: drugs, quantitative detection methods, and clinical efficacy and safety. In terms of drugs, the studies mainly cover four classes of anticancer therapies, namely: chemotherapeutic agents, endocrine therapy drugs, targeted therapy drugs and ICIs. Chemotherapeutic agents often exhibit high inter-individual PK variability, a well-established exposure-response relationship such as the correlation between area under the curve and pharmacodynamic endpoints, and sufficient time delay between sampling and clinical effect; these features collectively represent key criteria supporting TDM implementation (2). Busulfan remains the most studied agent in this class, particularly in hematopoietic stem cell transplantation settings (34–37), while methotrexate and 5-fluorouracil also demonstrate clear associations among drug exposure, efficacy, and toxicity (38–41). For TKIs, TDM is increasingly relevant because these drugs are metabolized by cytochrome P450 enzymes, are prone to drug–drug interactions, and exhibit plasma concentration variability driven by genetic polymorphisms that significantly affect clinical outcomes (42); imatinib, crizotinib, and osimertinib exemplify TKIs for which emerging evidence links specific PK parameters to improvements in survival outcomes (43–49). Monoclonal antibodies (mAbs) display more complex pharmacokinetics determined by target-mediated disposition, FcRn-mediated recycling, and inter-patient differences in target expression, yet trastuzumab and bevacizumab continue to appear frequently in TDM-related research (50, 51). TDM for endocrine agents such as tamoxifen is complicated by CYP2D6-dependent metabolic activation and inter-individual variation in metabolizer status (52–54). Similarly, for ICIs, inconsistent exposure-efficacy relationships, the absence of established therapeutic concentration ranges, and confounding effects of target binding currently limit the clinical utility of TDM (4, 51, 55).

Although preliminary pharmacology and pharmacokinetic studies on antibody-drug conjugates (ADCs) have emerged during our study period, and although ADCs are often termed “magic bullets” due to their precise tumor targeting and typically narrow therapeutic window, there remains no consensus on the optimal analyte(s) for clinically meaningful TDM (56). Accumulating evidence indicates that different analytes may differentially inform efficacy versus toxicity. The intact (conjugated) ADC, as the pharmacologically active species, shows stronger association with antitumor efficacy. In trastuzumab emtansine (T-DM1), higher exposure—particularly the trough concentration at cycle 1 day 21 (Cmin, C1D21)—was significantly correlated with longer progression-free survival (PFS) and overall survival (OS) in patients with HER2-positive metastatic breast cancer (57). Similarly, in trastuzumab deruxtecan (T-DXd), greater systemic exposure to the intact ADC was linked to higher objective response rates in early-phase trials (58). Conversely, released payload (e.g., DM1, DXd, MMAE) appears more predictive of toxicity. Elevated plasma concentrations of free MMAE were associated with increased risk of grade ≥2 peripheral neuropathy and neutropenia in patients receiving brentuximab vedotin (59). Likewise, higher systemic exposure to released deruxtecan (DXd) has been implicated in interstitial lung disease, a dose-limiting toxicity of T-DXd (58). In contrast, total antibody has not been clearly demonstrated to be consistently associated with efficacy or safety outcomes (60).

Given the increasing clinical use of bispecific antibodies (bsAbs), no TDM-related studies on bsAbs were identified in our bibliometric analysis, underscoring their limited representation in the current literature under our search criteria. Their unique mechanisms, such as dual-target engagement, target-mediated drug disposition, and high immunogenicity, pose distinct TDM challenges that differ from those associated with conventional monoclonal antibodies and ADCs (61–63). This underscores the need for future research to establish evidence-based TDM frameworks tailored to bsAbs.

In addition to drugs, we also identified research hotspots related to analytical methods, diseases and special populations. Detection methods such as liquid chromatography, mass spectrometry, LC-MS, and tandem mass spectrometry have been relatively maturely applied, while emerging technologies like electrochemical sensors (64) and biosensors (65) are beginning to emerge. A highly cited paper utilized paper spray mass spectrometry for the quantitative analysis of therapeutic drugs in dried blood spot samples, with the method capable of detecting sunitinib at extremely low concentrations (1 ng/mL) (66).Regarding diseases, the burst detection of disease-related keywords was largely associated with the use of specific drugs, reflecting that research in TDM is often driven by drug-specific therapeutic contexts. For example, bursts in “chronic myeloid leukemia” coincided with research on busulfan and imatinib, while “metastatic colorectal cancer” emerged alongside studies on 5-FU. Meanwhile, children/pediatric patients have consistently been a hotspot in TDM research for anticancer drugs, with the keyword remaining in a state of emergence over the long term. Since 2022, it has entered a strong emergence phase, reflecting a recent surge in studies focusing on pediatric populations. This growing attention highlights important opportunities for optimizing drug dosing in children, though challenges remain—such as limited PK data, ethical constraints in sampling, and variability in drug metabolism due to developmental changes.

The publishing and co-citation patterns of journals reflect the interdisciplinary nature of TDM research. Productive journals are primarily specialized in pharmaceutical and analytical sciences, whereas highly co-cited journals are rooted in clinical oncology and pharmacology. This divergence aligns with the dual-map overlay results and underscores that while TDM methodology is developed in technical disciplines, its impact is realized through high-impact clinical research.

From the publication volume by country and the international collaboration map, it can be observed that most of the countries with high publication volumes are major economies (67), a trend similar to that seen in the field of antimicrobial TDM research (10). Notably, despite the Netherlands not ranking among the top ten in overall GDP or per capita GDP (based on 2024 data from the International Monetary Fund) (67), it not only stands out in the research output and citation count within the field of TDM for anticancer drugs, but also has the strongest research intensity, indicating the country’s strong commitment and capability in this area. Indeed, according to a 2024 report by the Commonwealth Fund, an independent healthcare research organization, the Netherlands ranked second among high-income countries for healthcare system performance and topped the list for the best accessibility and availability of health services (68). While the goal of TDM is to achieve precision medicine, which aims for better clinical outcomes, it also signifies greater investment in health economics from both individual and national perspectives (69). Several European countries play significant roles in this field, with close inter-country collaborations and high RII. The United States has the highest publication and citation count for all its publications, which correlates positively with its high health spending (70) and reflects the global impact of their research. However, despite its high overall publication output, the country’s RII in research on anticancer drug TDM is not outstanding. Among the top ten institutions by publication volume, all are from France and the Netherlands, with each institution publishing at least 41 articles on average, 67.2 papers, and maintaining strong connections with other global institutions. Meanwhile, among the institutions or study groups, UNICANCER (the French national hospital network exclusively specialized in oncology) were found to have significantly higher research intensity than others, reflecting the strong focus of this French collaborative network. Notably, Slotervaart Hospital in the Netherlands was previously active in this field. However, the hospital ceased operations in 2018 due to financial difficulties. As a result, several leading researchers including Lankheet NAG and Huitema ADR moved to other institutions. Consequently, Slotervaart Hospital has had no recent research output in TDM.

**Figure 12:**
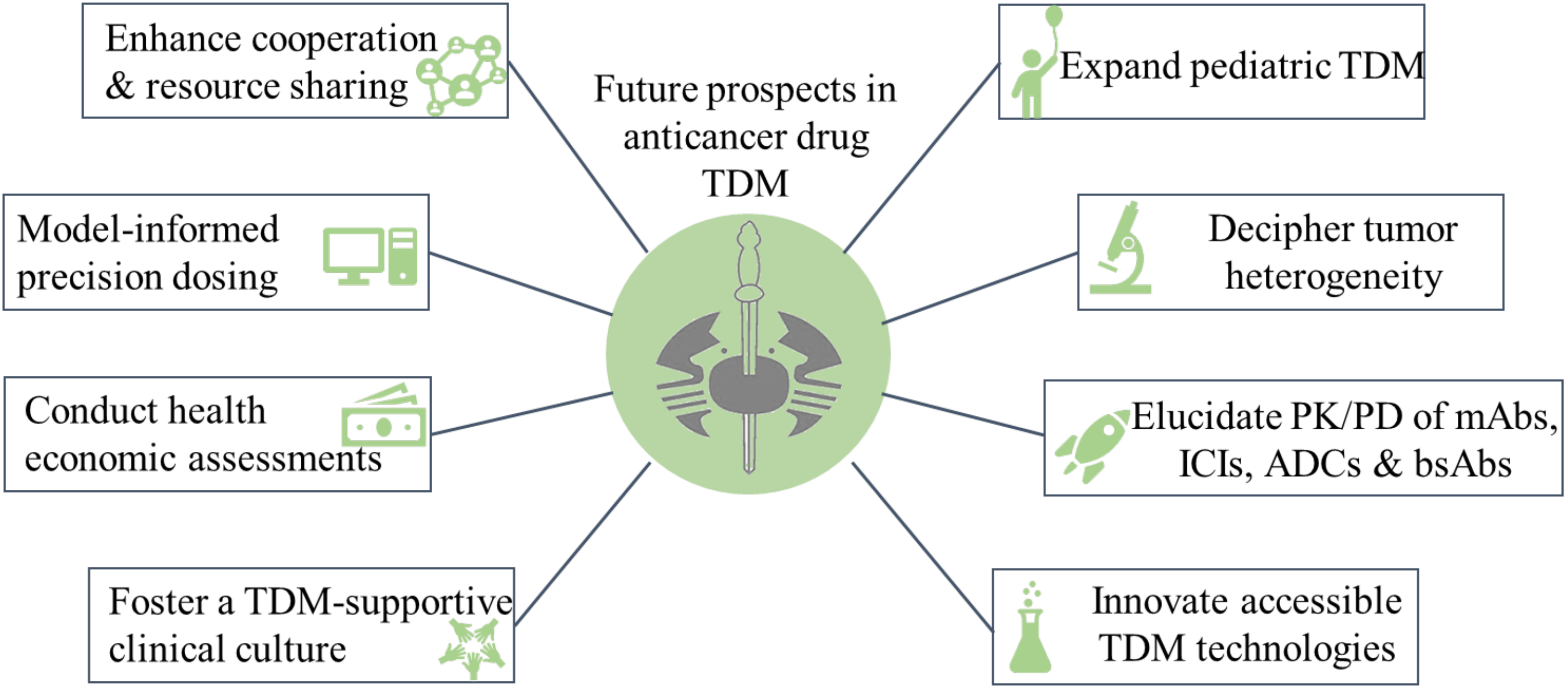
Future prospects in anticancer drug TDM.

Through bibliometric methods, we have identified and visualized several prominent keywords and research hotspots. However, some less explored areas can also be integrated with existing findings to collectively outline the future of TDM for anticancer drugs (**Figure 12**). Despite the current lack of widely accepted effective TDM strategies for pediatric cancer patients, further exploration in this field holds promise for significantly improving clinical outcomes and providing insights for personalized treatment in critically ill patients and the elderly (2). Additionally, deeper investigations into tumor heterogeneity are essential for better understanding drug mechanisms and individualized treatment needs (71, 72). On the pharmacological front, while some studies have examined the PK and PD of mAbs, many concentration-effect relationships have not been fully elucidated—particularly for ICIs, ADCs and bsAbs, whose multi-component exposure profiles, relevant biomarkers, and their links to efficacy and toxicity require systematic investigation (51, 56, 61, 73). In terms of analytical techniques, there is an urgent need to develop more precise, rapid, convenient, and cost-effective methods, such as biosensor-based platforms, alongside exploring alternative sample matrices like saliva, interstitial fluid, or dried blood spots to simplify collection processes (64, 74, 75). Future developments could include real-time visualization of test results via mobile applications, enhancing patient engagement and adherence, thus facilitating home-based TDM. Advanced modeling and simulation techniques should be introduced, leveraging high-quality clinical samples and incorporating machine learning algorithms to improve the predictive accuracy and personalization of TDM (76, 77). Furthermore, given the slowdown in global research progress over the past five years and insufficient investment by most countries, it is encouraged to strengthen international and institutional collaborations to share centralized laboratory resources and accelerate technological advancements. Simultaneously, fostering a supportive TDM medical culture is necessary, encouraging clinicians to adjust dosages based on monitoring results and urging pharmaceutical companies to address the complexities of personalized medicine (44). More high-quality clinical trials and prospective studies, especially Phase III trials, are critical for validating the effectiveness of new methods and technologies. Finally, health economic evaluations should be intensified, with national and governmental policy support needed to integrate TDM into healthcare systems, thereby enhancing its accessibility and prevalence (78). Achieving these goals requires the concerted efforts of clinicians, research institutions, pharmaceutical companies, regulatory bodies, and patients.

This study has several limitations. First, despite a relatively long search period, there is still a possibility of omissions, particularly as the analysis relied solely on the Web of Science database, which may introduce selection bias and limit comprehensiveness. Second, due to visualization requirements and threshold settings in bibliometric tools, not all keywords, institutions, or countries were fully represented, potentially leading to underrepresentation of weaker associations. Third, similar or misspelled terms—such as “tyrosine kinase inhibitor” and “tyrosine kinase inhibitors,” or “leukemia” and “leukeamia”—may have affected data accuracy. We attempted to merge these variants as much as possible, but some inconsistencies may remain due to the large volume of text. Additionally, bibliometric analysis cannot assess the qualitative depth of research or reflect actual clinical outcomes, so findings should be interpreted in conjunction with critical appraisal of study quality. Nevertheless, these limitations do not affect the overall conclusions of the article.

## 5 Conclusion

This study utilized bibliometric and visualization analysis methods to explore research trends and hotspots in TDM for anticancer drugs from 1990 to 2024, based on the WOSCC database. Over these 35 years, the number of papers on TDM for anticancer drugs has shown steady growth. Research primarily focused on drug exposure in special populations, hematologic diseases, TKIs, quantitative detection methods, chemotherapeutic drugs, and population pharmacokinetics. In the past three years, kinase inhibitors and pediatric patients have emerged as new research hotspots within the field. The thematic trend indicates a shift towards convergence from Molecular/Biology/Immunology and Health/Nursing/Medicine towards Medicine/Medical/Clinical. At the national level, the Netherlands and the United States have had significant impacts on this field, while at the institutional level, institutions from the Netherlands and France have contributed the most research outputs. Through this study, we hope to assist researchers in identifying new directions, hotspots, and frontiers in related areas.

## Data Availability

Publicly available datasets were analyzed in this study. This data can be found here: https://www.webofscience.com > wos Web of Science Core Collection.
